# The relationship between area levels of involuntary psychiatric care and patient outcomes: a longitudinal national register study from Norway

**DOI:** 10.1186/s12888-023-04584-4

**Published:** 2023-02-20

**Authors:** Olav Nyttingnes, Jūratė Šaltytė Benth, Tore Hofstad, Jorun Rugkåsa

**Affiliations:** 1 Health Services Research Unit, Akershus University Hospital, Nordbyhagen, Norway; 2 Centre for Research and Education in Forensic Psychiatry, Haukeland University Hospital, Bergen, Norway; 3 Institute of Clinical Medicine, University of Oslo, Oslo, Norway; 4 Centre for Medical Ethics, University of Oslo, Oslo, Norway; 5 Centre for Care Research, University of South-Eastern Norway, Notodden, Norway

**Keywords:** Involuntary care, Coercion, Compulsion, Severe mental disorders, Register study, Mental health legislation

## Abstract

**Background:**

Mental health legislation permits involuntary care of patients with severe mental disorders who meet set legal criteria. The Norwegian Mental Health Act assumes this will improve health and reduce risk of deterioration and death. Professionals have warned against potentially adverse effects of recent initiatives to heighten involuntary care thresholds, but no studies have investigated whether high thresholds have adverse effects.

**Aim:**

To test the hypothesis that areas with lower levels of involuntary care show higher levels of morbidity and mortality in their severe mental disorder populations over time compared to areas with higher levels. Data availability precluded analyses of the effect on health and safety of others.

**Methods:**

Using national data, we calculated standardized (by age, sex, and urbanicity) involuntary care ratios across Community Mental Health Center areas in Norway. For patients diagnosed with severe mental disorders (ICD10 F20-31), we tested whether lower area ratios in 2015 was associated with 1) case fatality over four years, 2) an increase in inpatient days, and 3) time to first episode of involuntary care over the following two years. We also assessed 4) whether area ratios in 2015 predicted an increase in the number of patients diagnosed with F20-31 in the subsequent two years and whether 5) standardized involuntary care area ratios in 2014–2017 predicted an increase in the standardized suicide ratios in 2014–2018. Analyses were prespecified (ClinicalTrials.gov NCT04655287).

**Results:**

We found no adverse effects on patients’ health in areas with lower standardized involuntary care ratios. The standardization variables age, sex, and urbanicity explained 70.5% of the variance in raw rates of involuntary care.

**Conclusions:**

Lower standardized involuntary care ratios are not associated with adverse effects for patients with severe mental disorders in Norway. This finding merits further research of the way involuntary care works.

## Background

Involuntary care is not uncommon in the treatment of persons diagnosed with severe mental disorders (SMDs). It remains a controversial aspect of psychiatric practice due to its obvious implications for patients’ autonomy and liberty. The use of involuntary care is regulated by mental health legislation, which specifies permitted forms of such care with criteria and safeguards. Involuntary care is usually restricted to those suffering from SMDs who are deemed to need involuntary care or considered a danger to themselves or others when there are no viable, less restrictive options [1]. The Norwegian Mental Health Act (NMHA) [2], like similar legislation in elsewhere, is founded on the assumption that involuntary care reduces risks to patients’ health or life or to the safety and lives of others [3].

Involuntary care is increasing in several countries, both in terms of inpatient [4, 5] and outpatient care [6–8]. The scientific evidence regarding benefits to patients of inpatient involuntary care is unclear. A limited number of effect studies, many of which suffered from methodological problems, used a multitude of relevant outcomes and showed mixed results [9–11], but overall, the benefits of involuntary admissions have not been firmly established. For Community Treatment Orders (CTOs), which oblige patients to adhere to treatment while living in the community, often with injections of long acting antipsychotic medication, there is a larger evidence base, and meta-analyses of effect studies have concluded that such orders do not prevent admissions or confer other patient benefits [12, 13]. A recent study indicated fewer suicides among patients under CTOs than among comparable patients discharged from hospital without a CTO [14].

Substantial variation has been observed in the use of involuntary care both between [4] and within [15, 16] jurisdictions. In Norway, a recent longitudinal population study found that the average rates of patients under involuntary care from 2014–2018 varied more than threefold between specialist Community Mental Health Center (CMHC) areas, while within-area rates remained relatively stable over time [17]. Age, deprivation, and urbanicity explain parts of the variance between areas, as shown in the United Kingdom [15], but a sizeable proportion of the variation remains unexplained, possibly reflecting differing thresholds for using involuntary care.

Observed variation in the use of involuntary care, along with persistent opposition to coercion by service user organizations, have triggered a number of policy initiatives, campaigns, and programs to reduce involuntary care in various countries [18–24]. Concerns have been raised, however, that a higher threshold for permitting involuntary care might have adverse effects on patients’ health by failing to alleviate heavy symptom burden or to reduce severe risks [25–28]. These seem to be relevant concerns, as many patients with psychotic disorders experience relapse [29], chronicity [30], and because patients with SMDs on average die 10–30 years younger than their peers [31]. We have not found empirical studies that have examined whether the lower use of involuntary care is associated with these forms of poor outcomes for patients.

### Aims

We present an analysis designed to address this gap in the literature. We hypothesize that if areas with lower levels of involuntary care do not achieve the legislative goals for involuntary care, this would, over time, manifest in higher levels of morbidity and mortality in the area’s population of people with SMDs compared to areas with higher levels of involuntary care. Specifically, we aim to answer the following research questions, using longitudinal national population data from Norway:

1. Do patients with SMDs who live in areas with a lower level of involuntary care have increased case fatality compared to those in areas with a higher level?
2. Do patients with SMDs who receive only voluntary care in the index year and live in areas with lower levels of involuntary care have a higher use of inpatient care in the two subsequent years compared to patients in areas with higher levels?
3. Do patients with SMDs who receive only voluntary care in the index period and live in areas with lower levels of involuntary care experience shorter times until an episode of involuntary care?
4. Does the number of patients with an SMD diagnosis increase over time in areas with lower levels of involuntary care?
5. Is the number of suicides per population higher in areas with lower levels of involuntary care compared to areas with higher levels?

## Method

### Setting

Norway is a sparsely populated country of five million inhabitants. Four Regional Health Authorities commission 21 health trusts to deliver specialist mental health care from hospitals and CMHCs. In 2016, 71 CMHC areas – the unit used in our analyses – ranged in size from 9,000–125,000 inhabitants aged 18 years and above. Their combined catchment areas covered the entire national population. The NMHA permits involuntary care for observation and treatment of both inpatients and outpatients [2]. A small number of patients are also committed to involuntary care by the criminal courts. Compared internationally, the Norwegian population rates of involuntary care are toward the higher end, with 151 involuntary hospitalized patients per 100,000 adult population in 2015 [4] and a point prevalence of 47.4 patients under a CTO per 100,000 adult population in 2012 [7]. In 2015, the review board rejected 66 of 7824 instances of initiated involuntary care after mandatory document control. In addition, 21 patients successfully appealed against an involuntary observation, and 156 against involuntary care [32].

### Study design and data

The study is a retrospective, longitudinal register study, with data from 2014–2018, where the involuntary care ratio in the area a patient lives is studied as a predictor of subsequent outcomes. We registered the analysis plan prospectively (Clinicaltrials.gov identifier NCT04655287).

We obtained patient data on patients aged 18–65 from the Norwegian Patient Registry (NPR), to which all Health Trusts are required to report all specialist inpatient or outpatient health service use. This means that those diagnosed with an SMD in one year but with no specialist service contact in the following year would not be counted for that second year. This registry provides reliable, patient-identifiable, national data with a good degree of completeness [33], including valid diagnoses for severe mental disorders [34]. From 2015 onward, data completeness for inpatient status (voluntary/involuntary) is considered adequate [35]. The *population at risk* for SMD and involuntary care was defined as all adults ages 18–65 in Norwegian municipalities and city districts (hereafter called local authorities), and data were acquired at Statistics Norway’s online table generator [36, 37]. We acquired data on suicides for men and women ages 18–65 years in CMHC catchment areas from the Norwegian Cause of Death Registry (NCDR). These could only be released aggregated to local authority level and could not be linked to individuals from the NPR data. Data availability precluded analyses of the effect on health and safety of others.

### Inclusion and exclusion criteria

The patient sample comprised all persons with an SMD diagnosis and all those with an episode of involuntary care (defined below) within the study period. We excluded those over 65 years to reduce a potential bias from involuntary care resulting (partly) from dementia.

Homeless persons (75 per 100,000 population) [38] were included as their home area is specified in the registers. We excluded persons without a Norwegian Identification number because they could not be followed over time or allocated to a CMHC area (ca. 1% of patients under involuntary care) and, for the same reason, those with unknown or missing data for residing local authority in 2015 (< 0.001% of persons under involuntary care).

### Variables

We based our operationalization of *severe mental disorder* (SMD) on the NMHA [39], which restricts the major diagnostic prerequisite for involuntary care to psychoses or psychotic symptoms. The law discourages involuntary care for mental health disorders without such symptoms [39]. Therefore, we defined an SMD as a diagnosis on the schizophrenia spectrum (F20-29) or bipolar disorder with psychosis (F30-31) as either the primary or secondary diagnosis, as classified following the ICD-10 system [40]. For patients with more than one recorded diagnosis during a year, we used the following hierarchical order to select each patient’s diagnosis: bipolar, schizophrenia spectrum, substance abuse (F10-19), personality disorder (F60-69), and depressive (F32) and other disorders.

For the purposes of the present study, *involuntary care* was defined as involuntary inpatient or outpatient care or observation sanctioned by the NMHA, including those in forensic care and those sentenced to care by a criminal court. A patient with such a care episode was classified as under involuntary care for that year.

*Mental health inpatient days* for a patient was the term we used for the combined number of days in hospital for that patient during the calendar year in question, regardless of care formality and including inpatient days in mental health institutions outside the patient’s CMHC area.

*Area of residence* represented the local authority in which the patient’s registered address belonged, aggregated to the appropriate CMHC area. For those who moved during the period, we used the address at the beginning of the last episode of mental health care during the year in question. For analyses that studied effects over time, the area of residence in the index year was used as the area of residency throughout.

*Urbanicity* was classified as one of five levels of urbanicity as described in Table 1, which were assigned to each local authority area. This was based on Statistics Norway’s classification [41] as modified in a previous study [42]. An urbanicity value was assigned to each patient based on his or her area of residence.

**Table 1:** Classification of urbanicity

Urbanicity level	Definition
1	Norway’s capital and four largest regional centers, as defined by Statistics Norway
2	Remaining local authorities with 20,000 + inhabitants, where 80% or more live in densely populated areas^a^
3	County centers in one of the 19 counties not classified as 1 or 2, plus urban local authority areas that are a continuation of a densely populated area with urbanicity level 1 or 2 (typically suburbs)
4	Remaining local authorities with 5,000 + inhabitants, of which 60–79% live in densely populated areas
5	Remaining local authority areas

^a^Statistics Norway defines a densely populated area as a collection of houses where at least 200 persons are living and the distance between houses does not exceed 50 m

*Area deprivation.* We used Statistics Norway’s continuous living condition index for 2008 as a measure of deprivation in the local community. The index is based on averaged deciles of unemployment, welfare benefits, educational level, mortality, etc., resulting in a number between 1 and 10 [43] and was updated every eight years until 2008.

*CMHC areas* consist of one or more local authorities. Several CMHCs and local authorities merged during the study period (2014–2018). In order to have a fixed area structure in the analyses, we analyzed all data using the area structure from 2016. In two cities, CMHC areas deviated from local authority borders, and in these two cases, we combined CMHC areas, thereby reducing the number of areas from 71 to 69.

The *standardized involuntary care area ratio (SIAR)* was used as the primary covariate in our analyses. First, raw involuntary care rates were calculated by dividing the number of people experiencing involuntary care during a given year by the population at risk during that year. We prepared the standardization by estimating a linear regression model with the raw rate of persons experiencing involuntary care in the local authority as the outcome and with age (six groups), sex (two groups), urbanicity (five classes), and deprivation (range) as covariates. We did not include SMD rates into the standardization to reduce the risk of bias from differing diagnostic thresholds. We reasoned that a lower threshold for setting SDM diagnoses would mean more patients with less severe symptoms – and therefore unlikely to be treated involuntarily – would be diagnosed. If used in standardization, areas with lower threshold for setting SMD diagnosis could therefore have their rate of involuntary care adjusted downwards, whereas for areas with a higher diagnostic threshold this would adjust their level upwards. If service capacity or paternalism is associated with the threshold for involuntary care and SMD diagnosis setting, including SMD rates in the standardization could bias the analyses in the direction of null-results.

The variables that were significant in the regression model, which were age, sex, and urbanicity, were then used for indirect standardization. For each local authority, we calculated the expected number of patients under involuntary care for each age and sex stratum in the area’s urbanicity and aggregated this to the CMHC area population. We then divided the observed number of persons under involuntary care by the expected number of persons under involuntary care. The resulting SIAR values (range 0.58–1.46) have 1 as the reference, where SIAR values below 1 indicate fewer persons than expected under involuntary care in the CMHC area. Each patient was assigned a SIAR value based on his or her area of residency in 2015.

### Statistical analyses

To answer our research questions about possible negative effects of low levels of involuntary care, we predefined five models [44] (see Table 2). The first four were estimated using longitudinal data at the patient (Models 1–3) or CMHC area level (Model 4). In Model, 5 which used area suicides as outcome, we aggregated suicide numbers for five years in order to increase power. It is therefore a cross-sectional analysis.

**Table 2:** Overview of statistical models for adverse effects of low standardized ratios of involuntary care^a^ in CMHC^b^ areas

Longitudinal data at patient level	Sample	Hypothesized outcome in CMHC^b^ areas with lower SIAR^a^ in the baseline year(s)	Statistical approach
Model 1 (Case fatality)	Individual patients with SMD^c^ in 2015	Increase in number of deaths among SMD^c^ patients, 2015–2018	Cox regression, with patient’s survival time as outcome, adjusted for age and sex
Model 2 (Inpatient days)	Individual patients with SMD^c^ and no involuntary care in 2015	Increase (or lower decrease) in the number of mental health inpatient days from 2015– 2016 and/or 2017	Linear mixed model with random effects for CMHC^b^ and change in inpatient days from 2015–2016 and 2015–2017 as outcome
Model 3 (Involuntary care)	Individual patients with SMD^c^ and no involuntary care in 2015	Increase in number of patients transitioning into involuntary care in 2016 and/or 2017	Cox regression, with death as a competing risk and random effects for CMHCs^b^, with time to an incident of involuntary care during the next two years as outcome
**Longitudinal data at the CMHC level**			
Model 4 (SMD^c^ patients)	CMHC^b^ areas in 2015	Increase in number of persons diagnosed with SMD^c^ in 2016 and/or 2017	Linear mixed model with time, standardized CMHC^b^ ratios of involuntary care in 2015 and interaction between the two as covariates; outcome was yearly number of patients with SMD^b^ in the area
**Cross-sectional data at the CMHC level**			
Model 5 (Suicides)	CMHC^b^ areas in 2014–2017^d^	Higher suicide ratios in 2014–2018	Correlation between mean of the standardized yearly ratios of involuntary care in 2014–2017 with the similarly standardized 5-year ratio of suicides in the CMHC^b^ areas in 2014–2018^d^

^a^CMHC area population ratios of involuntary care, standardized by age, sex and urbanicity of living area

^b^*CMHC* Community Mental Health Center

^c^*SMDs* Severe mental disorders, ICD-10 codes F20-31

^d^As suicides could not be allocated to some local authority areas, data were merged in 10 of the CMHC areas

Data were not available in advance to prespecify any cut-off value of high/low SIAR or to decide between non-linear and linear associations between SIAR and the outcomes. Therefore, we examined each outcome for non-linear associations and for adequacy of a linear model with suitable tests and estimated the latter when appropriate.

Model 1 – Cox proportional hazards (PHs) regression analysis. We assessed the effect of SIAR on case fatality over time, adjusted for age and sex, by following all SMD patients over four years, which allowed for observing delayed deaths. Schoenfeld’s residuals were used to assess the PHs assumption, while potential non-linear associations were tested by martingale residuals. Cluster effects on CMHC, Health Trust, and regional health authority levels were assessed by intra-class correlation (ICC), which showed no effects or negligible effects, and hence, no adjustment was needed. Models 2 and 3 assessed whether SMD patients in voluntary care in 2015 deteriorated over the subsequent two years in areas with low SIAR. As we here wanted test deterioration (into more inpatient days in Model 2 and into involuntary care in Model 3), we included only those in voluntary care in the baseline year. It is also among voluntary patients with SMD that we could expect to find those whose needs for protection against deterioration may not be met where there is a high threshold for using involuntary care. Model 2 was a linear mixed model, which assessed the trend in inpatient days as a function of SIAR. The model contained random intercepts for CMHCs and fixed effects for time dummy, SIAR, and interactions between time and SIAR. A model with SIAR as a non-linear covariate was considered; however, no non-linear associations were detected. The residuals were inspected graphically to assess the assumptions of normality and homoscedasticity. Post-hoc analyses were performed to explore the interaction further. Model 3 – a Cox PH model – assessed time to involuntary care as a function of SIAR. Death was included as a competing risk. The model assumptions were assessed in the same way as for Model 1.

Model 4 – a linear mixed model – assessed whether low SIAR in a CMHC area was followed by an increased number of people treated for SMDs in the area in the following two years, suggesting differential rates of improvement or deterioration. As this is an area characteristic it was analyzed at area level, and different baseline levels of SMDs were controlled for by investigating changes in SMD rates. The model included fixed effects for time as a second-order polynomial to account for non-linear effects, SIAR, and interaction between the two. The model without the interaction term was chosen based on the Bayesian information criterion (BIC). The model assumptions were assessed in the same way as for Model 2.

Model 5 was based on cross-sectional data for SIAR and suicides; scatter plot and correlation analysis was employed to assess whether a low SIAR is associated with more suicides. For the smallest CMHC areas, the expected number of suicides per year (based on the national count of ca. 600 suicides per year [45]) was around 1 per year. Therefore, we aggregated suicide numbers for 2014–2018 in order to increase power. This required a cross-sectional design, and we therefore observed and averaged involuntary care for the years 2014–2017, to observe suicides that might happen some time after discharge from involuntary care. We considered an area level measure of suicide to be a relevant outcome for the SMD population, even if it might introduce some noise in that it includes suicides that are not SMD related.

For all analyses, we set the threshold for significance at 0.05. We prepared data for analyses using R 3.6.1 [46] with data.table 1.12.8 [47], tidyverse 1.3.0 [48], and lubridate 1.7.4 [49], and we used Stata 16 for standardization and regression models. Figures were drawn with Stata, ggplot2 [50] and MATLAB R2020b.

### Changes to the analysis plan

For technical reasons, we needed to make minor alterations to the registered analysis plan for Model 5. Due to several municipality mergers, the NCDR could not map data on suicides from 2019 to the local authority structure of 2018 and earlier; therefore, we moved the periodization from the planned period of 2015–2019 to 2014–2018. In addition, because the NCDR could not allocate suicides to city districts, we merged several CMHCs in cities for the model with suicides as the outcome. Due to the registry’s privacy requirements, a minimum cell size was required in order to release area data for suicides per urbanicity stratum, and therefore, we combined level 4 (the smallest stratum) and level 5. In 2014, four health trusts had < 85% data completeness on care formality. Thus, we ran Model 5 with and without data from nine CMHC areas in these four health trusts.

### Ethics

The study analyzed data from the NPR that was collected prior to and independently of this study. The Norwegian Research Ethics Committee granted permission to obtain and analyze de-identified data from the NPR without individual consent (ref: 2018/795). The NPR de-identifies patients’ ID numbers before release in accordance with relevant regulations. The study and the Data Protection Impact Assessment (DPIA) were approved by the Privacy Ombudsman at Akershus University Hospital (ref: 2018–090).

## Results

In Table 3, we provide details of the 21,481 included patients in 2015. One-third (6,853 patients) experienced involuntary care that year, 5,096 (74.3%) of whom were diagnosed with SMD. Of the remaining patients, 621 (9.1%) were diagnosed with addiction disorders and 246 (3.6%) with personality disorders. For all variables with available data for patients with only voluntary care in 2015, the difference between this group and patients under involuntary care had *P*-values lower than 0.001. There were significantly more men than women under involuntary care, and more urban areas had higher proportions of patients under involuntary care.

**Table 3:** Characteristics of patients with severe mental disorders or an episode of involuntary care in Norway in 2015

Variable	All included patients in 2015^a^	Under involuntary care in 2015	No involuntary care in 2015^b^
N (%)	21 481 (100)	6 853 (31.9)	14 628 (68.1)
Age in years, mean (s.d.)^c^	40.2 (12.5)	39.7 (12.6)	40.4 (12.4)
Female, *n* (%)^d^	10 432 (48.6)	2 982 (43.5)	7 450 (50.9)
Male, *n* (%)^d^	11 049 (51.4)	3 871 (56.5)	7 178 (49.1)
**ICD 10 diagnosis, *n* (%)**			
F10-19	621 (2.9)	621 (9.1)	–
F20-29^d^	11 056 (51.5)	4 074 (59.4)	6 982 (47.7)
F30-31^d^	8 668 (40.4)	1 022 (14.9)	7 646 (52.3)
F32	203 (0.9)	203 (3.0)	–
F60-69	246 (1.1)	246 (3.6)	–
Other^e^	687 (3.2)	687 (10.0)	–
**Urbanicity, *n* (%)**^**d**^			
1	7 109 (33.1)	2 545 (37.1)	4 564 (31.2)
2	6 409 (29.8)	2 117 (30.9)	4 292 (29.3)
3	2 821 (13.1)	797 (11.6)	2 024 (13.8)
4	2 077 (9.7)	563 (8.2)	1 514 (10.4)
5	3 065 (14.3)	831 (12.1)	2 234 (15.3)
**Involuntary care status, *n* (%)**	**6 853 (31.9)**	**6 853 (100)**	**-**
Inpatient, *n* (%)	5 581 (26.0)	5 581 (81.4)	-
Outpatient,* n* (%)	3 283 (15.3)	3 283 (47.9)	-
Both forms,* n* (%)	2 011 (9.4)	2 011 (29.3)	-
Sentenced to involuntary care in criminal court, *n* (%)	178 (0.8)	178 (2.6)	-

^a^Patients were included in the sample if they were either diagnosed with F20-31 or exposed to involuntary inpatient or outpatient care or both

^b^Due to the inclusion criteria, data are not available for all variables

^c^Independent samples t-test *P*-value for difference between patients exposed vs not exposed to involuntary care in 2015 < .001

^d^χ2-test *P*-values for difference between patients exposed vs not exposed to involuntary care in 2015 < .001

^e^Among ‘other’ diagnoses, organic (F00-09) and neurotic (F40-48) disorders were most common

**Fig. 1:**
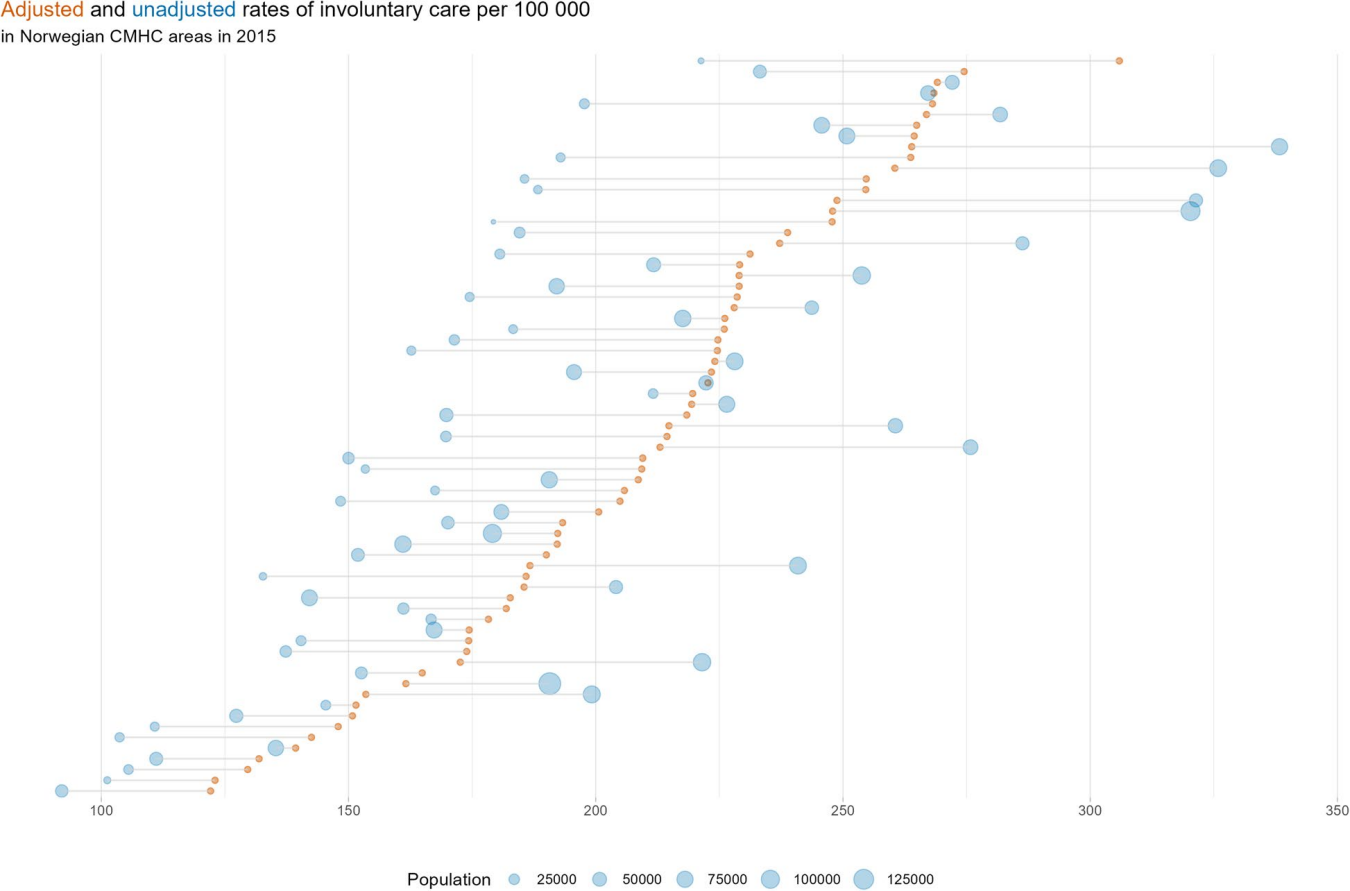
Illustration of involuntary care rates for adults (18–65) in community mental health center areas in Norway in 2015, as raw rates and standardized by age, sex and urbanicity (SIAR * national raw rate of 210.3). Lollipop lines for each CMHC area

The raw rate of persons under involuntary care in the CMHC areas varied from 92 to 338 per 100,000, with an extremal quotient of 3.7. In bivariate models, age explained 19.5% of this variance, sex 8.8%, urbanicity 42.1%, and deprivation 0.8%. The multiple regression model that included the statistically significant variables (age, sex, and urbanicity) as explanatory variables explained 70.5% of the variance in involuntary care between local authorities. In the indirect standardization, the SIAR in CMHC areas with a mainly urban population was lower compared to raw rates and vice versa. The raw and standardized rates for each CMHC area are shown in Fig. 1. The lowest and highest SIAR observed in 2015 was 0.58 and 1.46 (mean = 1.0), respectively, with an extremal quotient of 2.5.

### Models

In Model 1, we investigated case fatality for patients with SMDs dependent on SIAR. Of the 19,724 patients with SMDs in contact with specialist services in 2015, 739 died within the study period. The regression coefficients from the model with case fatality as the outcome are shown in Table 4. There was no significant effect of SIAR on case fatality. Men (*p* < 0.001) and older patients (*p* < 0.001) had a significantly higher rate of case fatality.

**Table 4:** Cox PG regression model for time to death for patients with SMD in 2015 who died between 2015 and 2018, with standardized ratio of involuntary care (SIAR) as primary covariate

Covariate	Bivariate models		Multiple model	
	**HR (95% CI)**	***p*-value**	**HR (95% CI)**	***p*-value**
SIAR^a^	0.88 (0.60; 1.28)	0.501	0.89 (0.61; 1.30)	0.538
Sex, male	1.51 (1.30; 1.75)	< 0.001	1.63 (1.40; 1.89)	< 0.001
Age (years)	1.06 (1.05; 1.07)	< 0.001	1.06 (1.05; 1.07)	< 0.001

^a^CMHC area population ratios of involuntary care, standardized by age, sex, and urbanicity of living area

In Model 2, we investigated the change in mental-health inpatient days for voluntary SMD patients dependent on SIAR. Of the 14,628 patients diagnosed with SMD and no involuntary care episodes in 2015, the mean number of mental-health inpatient days was reduced by one day per year, from 11.5 (SD 32.42) in 2015 to 10.5 (33.72) in 2016 and to 9.7 (32.93) in 2017. The model showed non-significant interactions, indicating no overall differences in the association between SIAR and the change in inpatient days, as shown in Table 5.

**Table 5:** Linear mixed model of change in mental health inpatient days from 2015 to 2016 and 2017 for patients with SMD and no involuntary care in 2015, with standardized ratio of involuntary care (SIAR) as the primary covariate

Parameter	Regression coefficient (SE)	*p*-value
Intercept	8.73 (1.45)	< .001
Year 2015 – ref	0	
Year 2016	-0.07 (1.65)	0.964
Year 2017	1.21 (1.65)	0.463
SIAR^a^	2.82 (1.44)	0.050
Year 2016 × SIAR^a^	-0.98 (1.64)	0.551
Year 2017 × SIAR^a^	-3.07 (1.64)	0.060

^a^CMHC area population ratios of involuntary care, standardized by age, sex, and urbanicity of living area

**Fig. 2:**
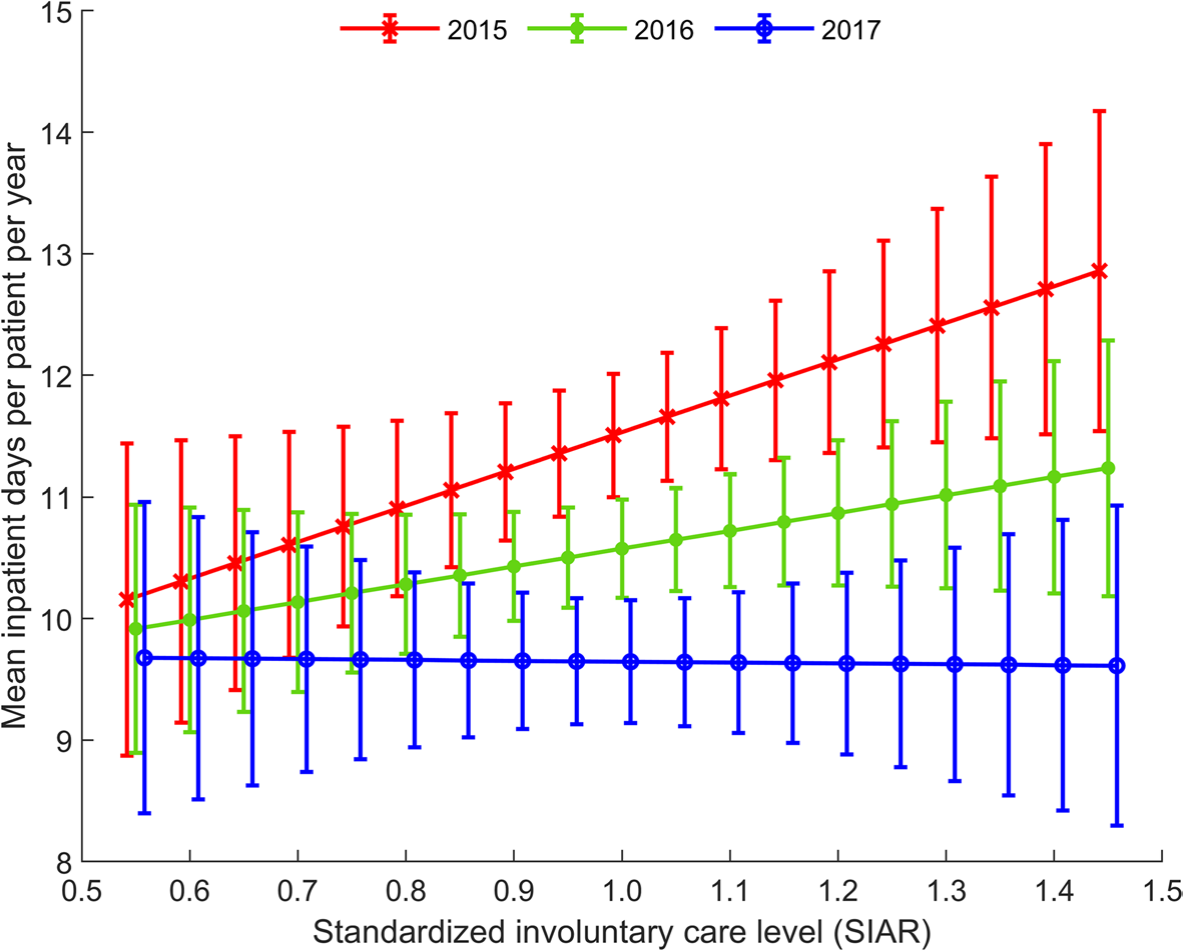
Predicted mean number of yearly inpatient days as a function of standardized (by age, sex, and urbanicity) involuntary care ratios for each of the three years with a 95% confidence interval

Because the *P-*value for SIAR (= 0.05) was close to our set significance level, we explored data in greater detail in a linear mixed model, showing a mean reduction of 0.92 days per year that was significant (*p* < 0.001). In this post hoc analysis, the change from 2015 to 2017 was significant for SIAR > 0.7, while the change from 2015 to 2016 was significant for SIAR values 0.85–1.35. The differences in change from 2015 to 2016 and 2015 to 2017, depicted in Fig. 2, are only significant, however, for SIAR > 0.9. Higher SIAR was associated with a higher number of inpatient days in the baseline year, and decreased more over time compared with lower SIAR areas.

In Model 3, we investigated time to involuntary care for voluntary SMD patients, dependent on SIAR. Of the 14,628 patients diagnosed with SMD and with no involuntary care episode in 2015, 1,202 (8.2%) experienced involuntary care at some point during the subsequent two years. The Cox PH regression model with death as a competing risk showed that lower SIAR in 2015 did not predict deterioration into involuntary care (hazard ratio 1.17, 95% CI (0.86; 1.57), *p* = 0.316). A sensitivity analysis that excluded patients who died showed similar results.

In Model 4, we investigated change in SMD rates in CMHC areas over time, dependent on SIAR. The rate of SMD per 100,000 persons across all CMHC areas varied between 606.5 in 2015 and 620 in 2017.

As presented in Table 6, the linear mixed model showed a significant non-linear time trend for the rate of SMDs, with an overall increase in rates throughout the period and a small reduction in 2018. Lower SIARs in 2015 were associated with lower SMD rates for 2016–2018 (*p* = 0.024), and this association did not vary between the years (no interaction between time and SIAR). The model predicted that areas with SIAR 1.1 had 2.5% more SMD patients per capita compared to areas with SIAR 1.0. That is, if an area has 20 additional people under involuntary care per 100,000 persons at risk, there would be 15 more people with an SMD in the area.

**Table 6:** Linear mixed model of number of SMDs in 2015–2018 for CMHC areas, with standardized ratio of involuntary care (SIAR) in 2015 as the primary covariate

Parameter	Regression coefficient (SE)	*p*-value
Intercept	0.004449 (0.00066)	< 0.001
Time	0.000224 (0.000078)	0.004
Time x time	-0.000060 (0.000025)	0.017
SIAR^a^	0.001463 (0.000648)	0.024

^a^CMHC area population ratios of involuntary care, standardized by age, sex, and urbanicity of living area

In Model 5, we studied suicide rate dependent on SIAR. We observed 2,474 suicides between 2014 and 2018, corresponding to a five-year suicide rate of 75.3 per 100,000 persons at risk (46.3 for women and 102.8 for men). The five-year rate varied by age from 65.7 in 18–25-year-olds to 92.2 for those ages 50–57. The correlation between standardized suicide rates for the five-year period and the mean of yearly local SIARs in the period 2014–2017 was -0.11, explaining 1.22% of the variance in suicides. After removing data from nine CMHC areas due to the reported low data completeness on legal status in 2014, the correlation coefficient was -0.15, explaining 2.3% of the variance. The scatter plots did not indicate non-linear relations.

## Discussion

We designed this study to investigate whether services in areas with lower ratios of involuntary mental health care fail to achieve legislative ambitions for involuntary care, specifically in regard to restoring health and reducing risks of deterioration or death for persons with SMD. To our knowledge, this is the first empirical study to investigate potentially adverse effects of lower levels of involuntary care within an entire jurisdiction.

Using a continuous, standardized measure of each area’s level of involuntary care (SIAR), we tested five models of possible adverse effects of a lower use of involuntary care and found no statistically significant effect for patients with an SMD. We did not observe significantly more case fatalities, deterioration into involuntary care, or increases in inpatient days for patients with SMDs, nor did we find increased rates of SMDs or notable increases in the numbers of suicides at the area level. In addition to investigating linear effects, we checked all outcomes for non-linear relationships to identify potential cut-off values for high vs low SIARs with differential effect on outcomes but found none. Overall, our findings did not support the hypothesis that areas with low standardized rates of involuntary care place patients with SMD at higher risk.

While none of our results reached statistical significance, the relationship between SIAR and number of inpatient days in Model 2 had a p-value of 0.05. An exploratory post-hoc analysis (depicted in Fig. 2) showed a higher mean number of inpatient days at baseline for voluntary SMD patients in higher SIAR areas. We used inpatient days as a proxy for severity of illness [51]. We had expected that a higher SIAR value reflected a lower threshold for involuntary care, and that voluntary SMD patients in these areas would have reduced illness severity compared to lower SIAR area patients. Contrary to this expectation, Fig. 2 indicated fewer inpatient days in lower SIAR areas and that this inpatient day level was stable throughout the three years.

If SMD patients in areas with low standardized involuntary care rates have unnecessary deterioration and that fewer recover, then the following increased morbidity should lead to more patients under involuntary care. Model 3 and 4 showed no such effect, which is in accordance with the relatively stable involuntary care rates over time at CMHC level [17] as well as health trust level in Norway [52–55].

The national rate of involuntary care in Norway is relatively high compared to many other Western countries [4]. Rates considered low in Norway may, therefore, not be considered as such in other jurisdictions. Cross-national comparisons of involuntary care are fraught with problems [56], and several factors might be conducive to higher reported levels of involuntary care in Norway. These include good quality registers, relatively few patients in forensic care, inclusion of forensic patients in several coercion estimates, strict conditions for converting a patient’s status from voluntary to involuntary care during an admission, and the guidance from the Directory of Health that patients in need of treatment but without capacity to make treatment decisions should be placed under involuntary care, even if they accept treatment, in order to protect their legal safeguards [39]. Although the range of raw rates in CMHC areas in the data in our study (92–338 per 100,000) covers the national average level of involuntary care in many jurisdictions, several relevant parameters may vary between countries, and studies of the effect of the level of involuntary care in different jurisdictions are merited.

### Model assumptions and future areas for research

Our design was based on two main assumptions. First, as implicit in the legislation, involuntary care protects patients from negative outcomes. Second, our standardization procedure made the CMHC areas comparable. However, the results indicate that one or both of these assumptions are inaccurate.

The assumption that involuntary care protects patients from negative outcomes is not supported by our findings, but neither can it be ruled out. Combined with systematic reviews that demonstrate a lack of evidence for patient benefits of involuntary care [11, 13], this raises the concern that involuntary care may not work entirely as intended by lawmakers and clinicians. This might be because predicting suicides and other rare events is notoriously difficult [57]. We know that previously committed patients have greater likelihood of later involuntary care, which may maintain the patterns of involuntary care without fully taking into account the patient’s symptoms or risks ([58] see Lived experience commentary by Olive & Nyikavaranda). Given the concerns voiced by user organizations [59], the ethical concern surrounding the provision of treatment without a person’s consent [60, 61], and over geographical variations in such treatment [62] there is an urgent need for additional studies that test core assumptions of mental health acts.

To establish comparable areas, we calculated standardized involuntary care area ratios (SIARs) that controlled for age, sex, and urbanicity (deprivation added no explanatory value) as these variables are known to be associated with levels of involuntary care [15, 58]. Our standardization variables explained 70.5% of the variation in rates of involuntary care in 2015, and urbanicity, in particular, explained the observed service variation. Associations with urbanicity have also been found in the Netherlands [63] and England [15], and it was associated with an elevated risk of psychosis in studies from Northern European settings in a recent review [64]. A number of other factors that have been associated with our outcomes are likely to correlate with urbanicity and/or deprivation and, therefore, are to some extent controlled for by our procedure. These include immigrant status, which has been associated with higher levels of SMDs [65]; the proximity to a hospital [66]; and the numbers of hospital beds [67] and of psychiatrists [68], which have been linked to increased admissions. Nonetheless, there may be other factors that could not be fully evaluated in our design that may have affected the comparability of the CMHC areas. These could include variance in morbidity and severity (case mix), care capacity, diagnostic threshold, service paternalism, help-seeking behavior, and SMD recovery rates, among other factors.

The results in Model 4 suggest that confounding factors might have been at play. We found higher rates of SMD patients in areas with higher SIARs and no interaction between SIARs and time. With a higher level of morbidity – which was also suggested by baseline inpatient day use in Model 3 – it is possible that the threshold for using involuntary care was similar across areas. Nevertheless, results from Model 4 indicated that, with an additional 20 patients under involuntary care, there would be 15 additional people with SMD. With roughly one-third of all SMD patients under involuntary care in any given year (see Table 3), as many as 60 additional patients should be registered with SMD to explain why 20 more patients are placed under involuntary care, indicating that high SIAR areas have a lower threshold for involuntary care, a very different case-mix, or differing diagnostic practices.

Future research should seek to control for additional variables related to service context that were not included in our data. We know that the organization and capacity of primary mental health care vary significantly among the CMHC areas in Norway [69], which might have an impact on the use of involuntary care [70]. The availability of comprehensive outpatient treatment approaches, such as assertive community treatment (ACT) or flexible ACT, could also have an impact. Studies of ACT, where it exists in Norway, have found reduced numbers and duration of episodes of involuntary care [71], in addition to improved interaction and rapport between patients and professionals [72, 73]. This is in line with the international evidence on ACT [74]. Common qualities in these types of services may, therefore, have an impact on both the local level of involuntary care *and* potential adverse patient outcomes and, thereby, could contribute to the lack of difference in outcomes between areas with high and low SIARs as observed in our study.

### Strengths and limitations

The study was based on comprehensive and robust longitudinal data encompassing an entire jurisdiction, collected independently of the research aims and with good completeness. All analyses were prespecified and conducted with only minor alterations as explained above. The findings across all models were consistent and supported the null hypotheses. The SIAR comprised persons affected by involuntary inpatient and/or outpatient care, thus reducing possible bias from individuals with many involuntary care episodes during a year and from local differences in substituting admissions with CTOs.

A main limitation of the present study is the observational design. While the hypothesis of adverse outcomes for patients in low SIAR areas did not bear out in our analysis, unmeasured variables may have confounded the results, as discussed above. Our data did not allow us to test a number of alternative predictions regarding other important potential benefits of involuntary care, most notably, risk to others. Additionally, that SIAR did not predict outcomes in our design, does not inform us of the outcomes for patient groups that tend to be under involuntary care also in the low SIAR areas.

We categorized local authority areas into five ordinal classes. These urbanicity classes explained as much as 42.1% of involuntary care rate variation. As the underlying urbanicity dimension is likely to be gradual, a continuous urbanicity index could have improved the design.

To calculate SIAR rates, we looked at persons under involuntary care, but in Models 1–4, we looked at the outcome for persons with SMDs. Of patients under involuntary care in 2015, one-fourth did not have a primary or secondary F20-31 diagnosis, and Models 1–4 did not evaluate the effect for these patients. Future studies should consider more fine-tuned analyses by calculating separate involuntary care rates for prespecified diagnostic groups or should distinguish between involuntary care following, for instance, drug-induced psychoses vs longer-term mental disorders. Norwegian mental health care differs from care in other countries and changes over time, which limits the generalizability of the present study to other contexts.

In conclusion, based on the assumption underpinning the NMHA that involuntary care confers patient benefits by improving health and reducing risk, we hypothesized that areas with lower use of involuntary care would show poorer outcomes than areas of higher use. We found no such effect. Low area rates were not associated with higher case fatality, deterioration into involuntary care, or more inpatient days, nor did we find increased rates of SMDs or suicides at the area level. Services with lower levels of involuntary care, therefore, seem to provide care for those with SMD diagnoses with similar results as areas with higher uses of compulsion. Our results show that it is possible to study specific hypotheses of how involuntary mental health care works with routinely collected register data. More research is needed to examine in greater detail whether or not mental health legislation protects life and health in the ways intended by its core assumptions.

## Data Availability

The full dataset analyzed during the current study is not publicly available due to data restrictions demanded by the NPR. Population count data and aggregated suicide data are available from the corresponding author upon reasonable request.
